# Why Did Most French GPs Choose Not to Join the Voluntary National Pay-for-Performance Program?

**DOI:** 10.1371/journal.pone.0072684

**Published:** 2013-09-09

**Authors:** Olivier Saint-Lary, Erik Bernard, Jonathan Sicsic, Isabelle Plu, Irène François-Purssell, Carine Franc

**Affiliations:** 1 University Versailles Saint-Quentin en Yvelines, Department of Family Medicine, Montigny le Bretonneux, France; 2 University Versailles Saint-Quentin en Yvelines, Department of Family Medicine, Montigny le Bretonneux, France; 3 CERMES3 - UMR 8211 - INSERM U988, Villejuif, France; 4 Forensic Medicine University Paris Descartes, Paris, France; 5 Prospere Team Research, Paris, France; University of Washington, United States of America

## Abstract

**Background:**

In 2009, a voluntary pay for performance (P4P) scheme for primary care physicians was introduced in France through the ‘Contract for Improving Individual Practice’ (CAPI). Although the contract could be interrupted at any time and without any penalty, two-thirds of French general practitioners chose not to participate. We studied what factors motivated general practitioners not to subscribe to the P4P contract, and particularly their perception of the ethical risks that may be associated with adhering to a CAPI.

**Method:**

A cross-sectional survey among French general practitioners using an online questionnaire based on focus group discussion results. Descriptive and multivariate statistical analyses with logistic regression.

**Results:**

A sample of 1,016 respondents, representative of French GPs. The variables that were associated with the probability of not signing a CAPI were “discomfort that patients were not informed of the signing of a P4P contract by their doctors” (OR = 8.24, 95% CI = 4.61–14.71), “the risk of conflicts of interest” (OR = 4.50, 95% CI = 2.42–8.35), “perceptions by patients that doctors may risk breaching professional ethics” (OR = 4. 35, 95% CI = 2.43–7.80) and “the risk of excluding the poorest patients” (OR = 2.66, 95% CI = 1.53–4.63).

**Conclusion:**

The perception of ethical risks associated with P4P may have hampered its success. Although the CAPI was extended to all GPs in 2012, our results question the relevance of the program itself by shedding light on potential adverse effects.

## Context

The application of pay for performance (P4P) in primary care has experienced strong development during the past decade [1]. The logic of these payment systems, often additional, is derived from standard economic theory: a rational economic actor is sensitive to appropriate external incentives (here financial incentives), and regulators are responsible for adjusting these incentives to change the behavior of targeted actors in the desired direction [2]. Therefore, the primary purpose of introducing economic incentives is to allocate additional compensation to physicians (typically general practitioners) in exchange for higher-quality practices as measured by various indicators [3]. Although these mechanisms are becoming increasingly widespread, their effectiveness or innocuousness is the subject of debate [4].

In the United Kingdom, P4P has been generalized to all general practitioners (GPs) since 2004 and currently represents nearly one-third of their remuneration. Outcomes are routinely measured using a set of indicators that was developed by the Quality Outcomes Framework [5], [6]. Other countries, such as the United States, Australia, New Zealand and Israel, have also adopted a similar compensation method [7]. As health systems vary in ways they finance health care consumption and/or they organize the provision of and access to care, the results of the incentives created by an additional P4P mechanism are difficult to transfer from one system to another. For example, most GPs in France as in the United States are paid on a fee-for-service basis, whereas the United Kingdom primarily uses capitation payments. Furthermore, French and American GPs operate in hospital-centered health care systems, whereas GPs in the United Kingdom have a central position because of the importance of Primary Care Trusts (PCT) [8]. But despite these differences, a common set of characteristics and of unexpected consequences related to P4P seems to exist [4]. While P4P is usually designed to improve identified measurable clinical outcomes, such a system is also known to generate in the same time perverse incentives leading, for instance, to the exclusion of specific groups of patients [9].

In France, a voluntary P4P system was proposed to GPs in 2009, organized by the Public Fund (National Social Security). GPs had the opportunity to sign a contract called the “Contract for Improving Individual Practices” (CAPI in French) through a mutual agreement with the Public Fund [10]. This system, which was based on a set of 16 indicators, covered three main fields: prevention and screening, chronic diseases and prescription optimization. The third field was primarily aimed to encourage the prescription of generic or less expensive drugs.

In France, negotiations between the Public Fund and the medical unions occur every five years in order to establish rules related to the private medical practices and especially to set prices (regulated ceiling prices). In 2009, against the advice of the unions, the Public Fund offered to GPs the opportunity to sign individually a CAPI. Fieldwork was made by Public Fund representatives (who represent the public insurer, visit GPs and advise them on their practices). The CAPI allowed doctors to receive a maximum annual bonus of 5,000 Euros (representing nearly 7% of average turnover), depending on their achievement of objectives. No sanctions were planned, and it was possible to depart from the program at any time upon written request. In France, unlike in any other country, P4P was first introduced on a voluntary basis but the design was rather standard. Note that the number of indicators and the amount involved were both rather low in the initial version.

One and a half years after its introduction, there were approximately 16,000 GPs who had signed the CAPI, representing more than one-third of the target population [11]. Regarding Unions' opposition and the tense relationship between doctors and Public Fund, a lower adhesion rate was expected, and so it was considered a relative success. Nonetheless, the reasons that could prevent doctors from signing such a contract also raised questions. More than half of GPs did not sign this contract, although it provided neither sanctions nor irreversibility, and could only have increased their incomes. Despite the relative success, it was decided in the new medical convention that took place in July 2011 that P4P would apply to all GPs starting January 2012, unless they explicitly refused the agreement. In this context, it seemed essential to study the nature of the obstacles that could have dissuaded GPs from signing such a contract prior to its generalization. Was the choice determined by the French institutional context characterized by the lack of trust of GPs in the Public Fund [12], fears about control or by standard resistance to change? [13] Another argument may have been the doctors' perception of ethical risks associated with P4P. Ethical risks are defined here as calling into question at least one of the four fundamental principles of medical ethics (autonomy, beneficence, non-maleficence, and justice) [14].

From a survey of GPs that was conducted in 2011, we studied what determined their choice not to sign the P4P contract and, more particularly, their perception of the ethical risks that may be associated with adhering to a CAPI.

## Methods

Using an online questionnaire, we conducted a national survey among French GPs currently practicing in France.

### Survey instrument

The questionnaire was designed by four authors from different specialties (two GPs, a statistician and a sociologist). It was based on the results from two previous focus groups [15]. The results of these focus groups led us to divide questions into four parts: characteristics of the respondents, knowledge of indicators and adherence, ethical considerations, payment and relation to the Public Fund (Attachment 1). The survey instrument contained 36 closed-ended questions, and 17 questions were mandatory. The modalities of the answers were variable; except for the questions related to the characteristics of the practitioners, 18 were yes/no questions, and 10 other questions were answered on a five-point Likert scale. The questionnaire was pre-tested on 12 GPs in order to check its comprehension and acceptability.

### Sample

The inclusion criterion was such that the participants in the sample must be GPs currently practicing in France. Considering the number of studied variables and given that approximately one-third of French GPs signed a CAPI, we wanted a sample of 300 GPs who decided to sign the CAPI, and thus sought to have a total sample of 1,000 practitioners were recruited by email using the French Society of General Medicine (SFMG) contact list that included nearly 6,000 email addresses without distinction between GPs, specialists, institutional contacts, trainers etc. We specifically requested that only GPs respond, so it was not possible to determine precisely the initial eligible population. Expecting 1,000 respondents, we had only to send 2 reminders in a 3 weeks period (from 12/04/2011 to 30/04/2011).

### Ethics Statement

The study protocol was approved by the ethics committee of the French Society of General Practice (SFMG in French).

### Statistical analyses

We first described the questionnaire answers using Chi-2 independence tests; we studied the links between CAPI non-signatories and individual characteristics such as the perception of ethical risks associated with a CAPI and the perceived quality of relationships with the Public Fund. Then, a multivariate analysis was performed using logistic regression. The dependent variable was CAPI non-adherence. A backward elimination procedure was used to construct our model, with a significance level of 5%. The following adjustment variables were introduced into the model: gender, group/solo practice, peer group participation and relationship with the Public Fund. Observations with missing data were fully considered and no imputation method was used. The model selection was confirmed using forward and stepwise procedures which produced identical results. We also evaluated the quality of the final model by calculating the concordance percentage between the model predictions and the observed data.

All statistical analyses were performed using Stata SE 11 software.

## Results

### Sample characteristics

Among the 1,214 GPs who answered the questionnaire, 198 respondents did not meet the inclusion criteria. Thus, the analysis relies on 1,016 GPs among whom 322 signed the P4P program contract (31.7%).

The mean age of the GPs was 53; there were three times more male physicians than female physicians and the majority of the participants practiced in urban areas (59%) and in group practices (60%). The characteristics of the respondents are summarized in Table 1.

**Table 1:** Description of the sample in terms of socio demographic characteristics (n = 1.016).

Characteristics	Total *(n)*	no P4P		P4P		*p*
		*n*	(%)	*N*	(%)	
**Gender (n = 1,016)**						0.72
Men	769	523	(68.0)	246	(32.0)	
Women	247	171	(69.2)	76	(30.8)	
**Age (n = 1,013)**						0.83
<55	502	344	(68.5)	158	(31.5)	
≥55	511	347	(67.9)	164	(32.1)	
**Length of installation (n = 999)**						0.87
<25 years	516	354	(68.6)	162	(31.4)	
≥25 years	483	329	(68.1)	154	(31.9)	
**Location (n = 975)**						0.42
Rural	595	402	(67.6)	193	(32.4)	
Urban	380	266	(70.0)	114	(30.0)	
**Activity mode (n = 1,016)**						0.68
Group	606	413	(68.2)	193	(31.8)	
Individual	392	267	(68.1)	125	(31.9)	
**Peer group participation** 1 **(n = 1,016)**						0.79
Yes	571	392	(68.7)	179	(31.3)	
No	445	302	(67.9)	143	(32.1)	
**Intern supervisor (n = 1,016)**						0.58
Yes	432	291	(67.4)	141	(32.6)	
No	584	403	(69.0)	181	(31.0)	
**Quality of the relationship with the Public Fund (n = 1,000)**						<0.001
Bad	182	146	(80.2)	36	(19.8)	
Neutral	405	284	(70.1)	121	(29.9)	
Good	413	254	(61.5)	159	(38.5)	
**Total (%)**	**1 016**	**694**	**(68.3)**	**322**	**(31.7)**	

1 *A ‘Peer Group’ is constituted by 5 to 12 GPs practicing in the same area who meet regularly to exchange on their practices.*

### Description of results

#### Univariate analysis

Except for the perceived quality of their relationships with the Public Fund, the socio-demographic characteristics of the GPs were not associated with non-adherence to a CAPI (Table 1). In contrast, the perception of potential ethical risks associated with a CAPI appeared to be significantly associated with non-adherence: the GPs who decided not to sign a CAPI reported perceiving greater risks (Tables 2 and 3).

**Table 2:** Relationship between the CAPI subsciption and GP perception of ethical risks.

Characteristics	Total*(n)*	no P4P		P4P		*p*
		*n*	(%)	*N*	(%)	
**Decreases a patient's confidence in his or her doctor (n = 943)**						<0.001
Agree or rather agree	305	273	(89.5)	32	(10.5)	
Intermediate	173	141	(81.5)	32	(18.5)	
Disagree or rather disagree	465	213	(45.8)	252	(54.2)	
**Decreases patient autonomy (n = 924)**						<0.001
Agree or rather agree	266	234	(88.0)	32	(22.0)	
Intermediate	273	143	(52.4)	30	(47.6)	
Disagree or rather disagree	485	234	(48.2)	251	(51.8)	
**Increases GP authoritarianism (n = 973)**						<0.001
Agree or rather agree	600	485	(80.8)	115	(19.2)	
Intermediate	169	84	(49.7)	85	(50.3)	
Disagree or rather disagree	204	86	(42.2)	118	(57.8)	
**Decreases GP autonomy (n = 982)**						<0.001
Agree or rather agree	600	506	(84.3)	94	(15.7)	
Intermediate	147	84	(57.1)	63	(42.9)	
Disagree or rather disagree	235	74	(31.5)	161	(68.5)	
**Causes selection of the most adherent patients (n = 959)**						<0.001
Agree or rather agree	526	455	(86.5)	71	(13.5)	
Intermediate	145	98	(67.6)	47	(22.4)	
Disagree or rather disagree	288	89	(30.9)	199	(69.1)	
**Causes exclusion of the poorest patients(n = 950)**						<0.001
Agree or rather agree	423	376	(88.9)	47	(11.1)	
Intermediate	126	95	(75.4)	31	(24.6)	
Disagree or rather disagree	401	161	(40.1)	240	(59.9)	
**Generates new conflicts of interest (n = 965)**						<0.001
Agree or rather agree	631	547	(86.7)	84	(13.3)	
Intermediate	115	53	(46.1)	62	(53.9)	
Disagree or rather disagree	219	48	(21.9)	171	(88.1)	
**Total (%)**	**1 016**	**694**	**(68.3)**	**322**	**(31.7)**	

*Note: Missing data are not detailed (no significant difference between GPs who decided to sign the CAPI and those who decided not to sign the contract.)*

**Table 3:** Relationship between the CAPI subscription and opinions regarding this contract.

Opinion	Total	CAPI −		CAPI +		*p*
		*n*	(%)	*n*	(%)	
**A patient should be informed of whether his or her GP signs**						<0.001
Yes	628	543	(86.5)	85	(13.5)	
No	166	49	(29.5)	117	(70.5)	
No Decision	221	101	(45.7)	120	(54.3)	
**P4P reflects the financial quality of practices**						<0.001
Yes	1 41	39	(27.7)	102	(72.3)	
No	601	482	(80.2)	119	(19.8)	
No Decision	274	173	(63.1)	101	(36.9)	
**P4P can be perceived by patients as a breach of professional ethics by GPs**						<0.001
Yes	554	479	(86.5)	75	(13.5)	
No	198	59	(29.8)	139	(70.2)	
No Decision	264	156	(59.0)	108	(41.0)	
**The relatively small amount of P4P minimizes the risk of drift**						<0.001
Yes	289	126	(43.6)	163	(56.4)	
No	201	170	(84.6)	31	(15.4)	
No Decision	526	398	(75.7)	128	(24.3)	
**P4P threatens the dominance of the fee-for-service system**						<0.001
Yes	400	321	(80.3)	79	(19.7)	
No	600	363	(60.5)	237	(39.5)	
**P4P returns render doctors as similar to employees evaluated based on quantified targets**						<0.001
Yes	553	466	(84.3)	87	(15.7)	
No	237	99	(41.8)	138	(58.2)	
No Decision	226	129	(57.1)	97	(42.9)	
**The CAPI is able to assess the quality of practice**						<0.001
Agree or rather agree	82	23	(28.0)	59	(72.0)	
Intermediate	232	92	(39.7)	140	(60.3)	
Disagree or somewhat disagree	597	478	(80.0)	119	(20.0)	
**Do you know the indicators that are used in the CAPI?**						<0.001
Yes	507	257	(50.7)	250	(49.3)	
Intermediate	204	157	(77.0)	47	(23.0)	
No	280	257	(91.8)	23	(8.2)	
**Total (%)**	**1 016**	**694**	**(68.3)**	**322**	**(31.7)**	

#### Multivariate analysis

Four perceived ethical risks were significantly associated with a greater probability of not signing a CAPI: first, the perceived discomfort with the fact that patients were not informed of whether their GP has signed a CAPI or not (OR = 8.24; 95% CI = 4.61 to 14.71); second, the potential occurrence of new conflicts of interest (OR = 4.50, 95% CI = 2.42 to 8.35); third, the potential interpretation by patients that the physician has breached professional ethics (OR = 4.35, 95% CI = 2.43 to 7.80) and finally, the risk of excluding the most vulnerable patients (OR = 2.66, 95 = 1.53 to 4.63%).

Conversely, the following variables decreased the probability of failing to sign and thus favored the signing of a P4P: considering that a low premium amount could minimize the risk of adverse events (OR = 0.38, 95% CI = 0.19 to 0.76) and viewing the P4P as a reflection of the quality of medical practice (OR = 0.31, 95% CI = 0.16 to 0.61).

Among the studied socio-demographic characteristics age had a nonlinear effect (OR = 0.61, 95% CI = 0.45 to 0.82): young GPs and those over 60 refused to sign a CAPI more often than GPs between the age of 45 and 60.

Similarly, knowledge of the indicators decreased the probability of not signing a CAPI (OR = 0.09, 95% CI = 0.05 to 0.18).

The other included variables were not significantly associated with non-adherence to a CAPI.

With a pseudo *R^2^* of 0.487 and a percentage of agreement equal to 92.9% (Table 4), the model showed a good fit and a good predictive ability.

**Table 4:** Multivariate analysis: variables significantly associated with the CAPI subscription.

Variables and modalities	Adjusted odds ratio	(95% CI)	*p*
**GP characteristics**			
Age	0.61	(0.45–0.82)	0.001
Age^2^	1.00	(1.00–1.01)	0.004
Gender *(ref Male)*			
Female	0.66	(0.39–1.13)	0.128
Group practice *(ref Yes)*			
No	0.89	(0.56–1.42)	0.633
Peer group *(ref Yes)*			
No	0.79	(0.50–1.24)	0.302
Relationship with the Public Fund *(ref Good)*			
Neutral	0.85	(0.52–1.37)	0.500
Bad	0.89	(0.47–1.68)	0.711
P4P reflects the financial quality of practices *(ref No)*			
Yes	0.31	(0.16–0.61)	0.001
No Decision	0.86	(0.52–1.43)	0.568
Knowledge of the indicators that are used in the CAPI *(ref No)*			
Yes	0.09	(0.05–0.18)	<0.001
Intermediate	0.24	(0.12–0.51)	<0.001
**Ethical risks**			
A patient should be informed of whether his or her GP signs *(ref No)*			
Yes	8.24	(4.61–14.71)	<0.001
No Decision	1.42	(0.76–2.66)	0.274
P4P can be perceived by patients as a breach of professional ethics by GPs *(ref No*			
Yes	4.35	(2.43–7.80)	<0.001
No Decision	1.63	(0.90–2.97)	0.106
The relatively small amount of P4P minimizes the risk of drift *(ref No)*			
Yes	0.38	(0.19–0.76)	0.006
No Decision	0.79	(0.40–1.56)	0.495
P4P can lead to the exclusion of the most precarious patients *(ref Disagree or rather disagree)*			
Agree or rather agree	2.66	(1.53–4.63)	<0.001
Intermediate	1.49	(0.77–2.86)	0.233
P4P can lead to new conflicts of interest *(ref Disagree or rather disagree)*			
Agree or rather agree	4.50	(2.42–8.35)	<0.001
Intermediate	1.76	(0.87–3.54)	0.116

*Note: ref = reference category.*

## Discussion

### Statement of principal findings

To our knowledge, this paper describes the first study that uses the identification of the individual characteristics of GPs to explain their decision to enroll or not in a voluntary P4P scheme. Our database is a representative sample of more than 1,000 French GPs in terms of age (53 vs. 52 years), sex ratio (more than 75% of males), installation rates in group practices (60% vs. 54%) [16] and rates of P4P adherence [17]. Our findings identify two profiles of GPs: those perceiving ethical risks as low and agreeing to sign (31.7%) *versus* those perceiving such risks as rather high and deciding not to sign (68.3%). The lack of patient information regarding the status of their physicians is the main perceived risk reported by non-signatories. Other ethical risks associated with non-adherence include the occurrence of new conflicts of interest, the perception by patients of a breach of professional ethics and the possibility of excluding the most vulnerable patients. The context of our study is original, as the optional characteristic of the French P4P allows us to compare doctors' decision to join a national P4P program or not and thus, to reveal their “preferences” (in the economic sense).

Although French previous descriptive studies did not identify significant differences between GPs enrolled or not (in terms of age, gender, practice location and setup time) [18], the inclusion in our model of perceived ethical risks associated with P4P reveals different physicians' profiles. While gender and location had no significant effect in previous studies, in our model age is significantly associated with the P4P adherence decision. The nonlinear effect of age is such that younger physicians and older physicians appear to be more reluctant to engage in P4P. The CAPI occurred in a political context in which the often-mentioned obstacle involved a poor relationship between the GPs and the Public Fund. However, multivariate analysis controlling for the perception of ethical risks does not support this common assumption: the relationship between physicians and the Public Fund does not explain the decision not to sign the P4P contract.

### Strengths and weaknesses of this study

Our approach is original because it addresses the doctors' perception of potential ethical risks associated with P4P. In addition to showing that the perception of ethical risks is a barrier to the decision to engage in a voluntary based P4P contract, our study allows for the first time to prioritize these perceived risks. Predictably, all ethical risks appear to have a greater specific effect in the univariate analysis for physicians who have not joined the P4P. In the multivariate analysis, the logistic regression model demonstrates that five ethical risks remain significantly associated with non-adherence, despite the existence of a strong colinearity among these variables. These ethical risks remain the same regardless of the method used for variable selection (backward, stepwise); thanks to the size of the sample (more than 1,000 GPs), we are able to confirm the consistency and robustness of the model.

Due to our recruitment method of using the contact list of a scientific society of general medicine, nearly 40% of respondent doctors are intern supervisors (vs. 10% in France) [19]. We do not believe that this parameter affects the findings though, because the responses of intern supervisors and non-supervisors did not differ significantly.

The design of our study did not enable us to identify causal relationship; for example, we demonstrate that GPs' good knowledge of the indicators is associated with P4P adherence (the probability of not having signed the CAPI is significantly lower); but this variable might be endogenous because knowledge of the indicators may result from recently signing and already receiving information from the funder.

Moreover as our analysis focused on declarative responses to closed questions, our results are more likely to address stated perceptions than ‘true’ perceptions and this could limit the expression of certain views. However, this questionnaire was based on the results of a qualitative study [15], so it is reasonable to assume that the proposed questions provided an accurate representation of all of the common ethical risks perceived by French GPs.

### Explanations and implications in the French context

French GPs are primarily compensated by a fee-for-service (FFS) system, which also raises ethical issues, such as “induced demand” [20], [21]. However, French patients are still allowed to change GPs fairly easily, although in 2010, 92% of patients chose a GP as their “primary doctor” [22]. This high rate of declaration is primarily explained because patients' consultations (with GPs but also with specialists) are better reimbursed if patients reported a “primary doctor”.

French patients were not informed of the existence of the P4P and even less of the signature of the contract by their doctor. There was no obligation for GPs to inform patients, neither on their participation nor on their level of performance

All of these factors could influence the manner in which French GPs perceive the national P4P program.

Among the perceived ethical risks, patient information was the major barrier for the GPs who decided not to sign the P4P contract; conversely, those who decided to sign believe that it is not necessary to inform patients about their commitment to P4P. The French P4P program promotes the principle of beneficence by inciting physicians to follow national guidelines based on the best scientific evidence. However, the CAPI, as any other P4P program, may call the autonomy principle into question by encouraging physicians to focus on indicator objectives at the expense of meeting patient expectations.

### Comparison with international literature

At the international level, several publications have focused on the ethical risks of P4P, primarily through expressions of opinions [23]–[25], qualitative studies [15], [26] or studies that focused on only one adverse effect, such as, for example, the risk of reduced access to health care for minorities [27].

P4P might also bring a decline in personal/relational continuity of care between doctors and patients [28] mainly because P4P has undermined patient-centered care within consultations [29].

Other publications focused on the nature of barriers to make a P4P system acceptable, in other words, to maximize social acceptability. For instance resistance to change is generally described as a classical barrier to new remuneration schemes [13]

However, to the best of our knowledge, no previous work has prioritized the relative importance that GPs attach to each of ethical issues linked with P4P.

### Unanswered questions and future research

The extension of P4P to all GPs in France has been effective since January 2012, unless the specifically opt out. Our results highlight the importance of doctors' perception of the ethical risks that may be associated with P4P as a major barrier to adhering to such a payment system. It seems inevitable that some GPs experience “ethical conflicts”. The weight of the ethical tensions that they are forced to accept could have negative effects, for example, on their intrinsic motivations [30]–[32]. Although there is no consensus in the literature on this issue [33], some results tend to reinforce the hypothesis of the “crowding out” of intrinsic motivations by extrinsic motivations [34]. This effect is not likely to reduce the effectiveness of P4P from the regulator/payer's point of view, *i.e.* in terms of improvement of clinical outcomes, but it could have negative consequences on the quality of care and the satisfaction of doctors [35]. These kinds of potential consequences would certainly reduce the expected effectiveness of P4P in a collective point of view.
